# Hyperthermia Induces Apoptosis through Endoplasmic Reticulum and Reactive Oxygen Species in Human Osteosarcoma Cells

**DOI:** 10.3390/ijms151017380

**Published:** 2014-09-29

**Authors:** Chun-Han Hou, Feng-Ling Lin, Sheng-Mon Hou, Ju-Fang Liu

**Affiliations:** 1 Department of Orthopedic Surgery, National Taiwan University Hospital, Taipei 100, Taiwan; E-Mail: chhou@ntu.edu.tw; 2 Department of Dermatology, Sijhih Cathay General Hospital, Taipei 221, Taiwan; E-Mail: lavande213@gmail.com; 3 Department of Orthopedic Surgery, Shin-Kong Wu Ho-Su Memorial Hospital, Taipei 111, Taiwan; 4 Central Laboratory, Shin-Kong Wu Ho-Su Memorial Hospital, Taipei 111, Taiwan

**Keywords:** osteosarcoma, hyperthermia, endoplasmic reticulum (ER) stress, reactive oxygen species (ROS)

## Abstract

Osteosarcoma (OS) is a relatively rare form of cancer, but OS is the most commonly diagnosed bone cancer in children and adolescents. Chemotherapy has side effects and induces drug resistance in OS. Since an effective adjuvant therapy was insufficient for treating OS, researching novel and adequate remedies is critical. Hyperthermia can induce cell death in various cancer cells, and thus, in this study, we investigated the anticancer method of hyperthermia in human OS (U-2 OS) cells. Treatment at 43 °C for 60 min induced apoptosis in human OS cell lines, but not in primary bone cells. Furthermore, hyperthermia was associated with increases of intracellular reactive oxygen species (ROS) and caspase-3 activation in U-2 OS cells. Mitochondrial dysfunction was followed by the release of cytochrome c from the mitochondria, and was accompanied by decreased anti-apoptotic Bcl-2 and Bcl-xL, and increased pro-apoptotic proteins Bak and Bax. Hyperthermia triggered endoplasmic reticulum (ER) stress, which was characterized by changes in cytosolic calcium levels, as well as increased calpain expression and activity. In addition, cells treated with calcium chelator (BAPTA-AM) blocked hyperthermia-induced cell apoptosis in U-2 OS cells. In conclusion, hyperthermia induced cell apoptosis substantially via the ROS, ER stress, mitochondria, and caspase pathways. Thus, hyperthermia may be a novel anticancer method for treating OS.

## 1. Introduction

Osteosarcoma (OS) is the most common type of bone cancer characterized by locally aggressive growth and early metastatic potential [1]. Although OS is a relatively rare form of cancer, it is the most commonly diagnosed bone cancer in children and adolescents [2]. In recent decades, the combination of surgical resection and chemotherapy has improved the five-year survival rate [3]. Nevertheless, these treatments can cause side effects and induced drug resistance in OS [4]. An effective adjuvant therapy was insufficient for treating OS. Therefore, researching novel and adequate remedies is critical.

Accumulated evidence has suggested that hyperthermia can induce apoptosis in normal and tumor cells [5,6]. Normal tissues subjected to hyperthermia are accompanied by some of the normal physiological responses, such as microvascular expansion, a rapid breathing, a rapid heartbeat, increased cardiac output, increased blood flow, and increased kidney manufacturing of urine to dissipate heat [7]. Nevertheless, the damage caused by hyperthermia is not obvious in normal tissues. In cancer tissues, the blood vessels have an irregular architecture [8]. The microvascular permeability and blood circulation differ from those of normal tissues as the temperature increases, and damage becomes markedly obvious [9]. Moreover, a high temperature can denature protein, DNA, and RNA damage and interrupt vital cellular processes, ultimately causing cell death [10]. Previous studies have reported that hyperthermia is a suitable candidate for cancer therapy. Shellman (2008) stated that hyperthermia may be a promising treatment for basal cell carcinoma and melanoma by eliciting the intrinsic and extrinsic apoptosis pathways [11]. In 2013, Pawlik *et al.* provided evidence suggesting that one mode of heat-induced cell death in H1299 cells was mitotic catastrophe, which probably caused apoptosis [12].

Hyperthermia can induce endoplasmic reticulum (ER)-triggered apoptosis in numerous cancers, such as breast cancer, melanoma, skin cancer, OS, colon cancer and lung cancer [11,12,13]. The ER is responsible for protein modifications, protein folding, protein synthesis, and lipid synthesis. When cells are exposed to various stimuli (oxidation, heat, drug, damage, or infection), the ER homeostasis is disrupted, and unfolded or misfolded proteins accumulate in the ER. Cells then activate several signaling pathways, including the unfolded protein response (UPR) or ER-associated protein degradation [14]. These responses protect cells, but intense ER stress eventually causes cell apoptosis [15]. The ER chaperone proteins glucose-related protein 78 (GRP78)/Bip and GRP94, are the marker and key regulators of ER stress [16]. The GRP78 protein has anti-apoptotic properties, and can attenuate the UPR [17]. In addition, hyperthermia induces reactive oxygen species (ROS) and the functional disorders of the mitochondria in various cancer cell lines [18,19,20]. The ROS and mitochondria dysfunction play vital roles in the apoptotic process.

In this study, we demonstrate that hyperthermia markedly increased the cytotoxicity on OS cell lines. For the first time, we observed that hyperthermia activated ROS, mitochondria dysfunction, and ER stress, thereby activating caspase-dependent apoptotic pathways.

## 2. Results

### 2.1. Hyperthermia Induced Apoptosis in Human Osteosarcoma Cells

**Figure 1:**
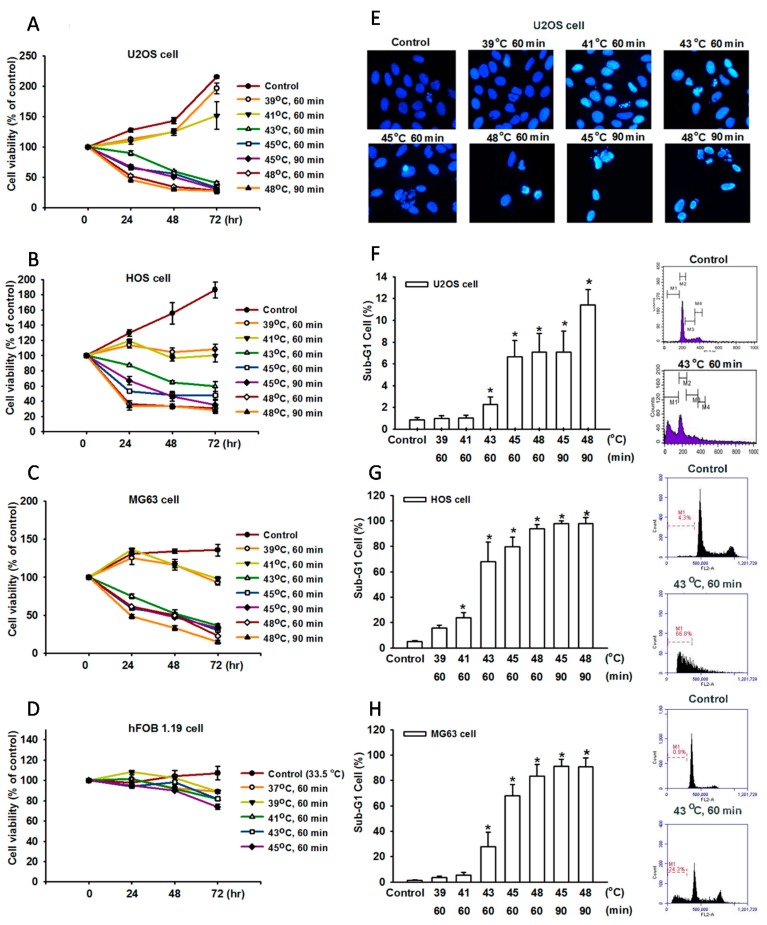
Hyperthermia-induced cell apoptosis in human OS cells. (**A**–**D**) Cells were incubated at various temperatures of hyperthermic conditions. After 24, 48, and 72 h, cell viability was examined by sulforhodamine B (SRB) assay; (**E**) U-2 OS cells were treated at various temperatures of hyperthermic conditions. After 24 h, DAPI stains was determined using immunostaining and were photographed using a fluorescence microscope; (**F**–**H**) of apoptotic cells was analyzed by conducting flow cytometric analysis on PI-stained cells. Cells were treated at various temperatures of hyperthermic conditions. After 24 h, the percentage. The results are expressed as the mean ± SEM of 4 independent experiments. *, *p <* 0.05, compared with the control group.

**Figure 2:**
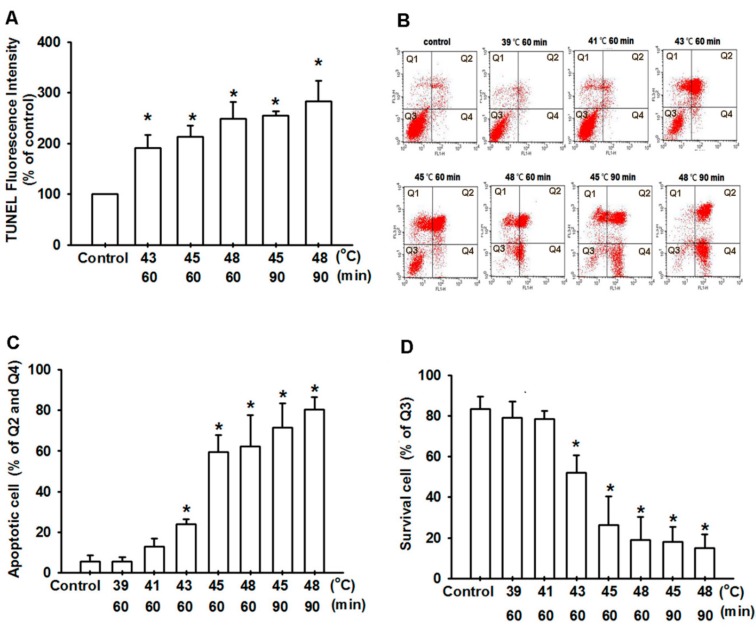
Hyperthermia-induced apoptosis of human OS cells. (**A**) Cells were treated at various temperatures of hyperthermic conditions. After 24 h, TUNEL-positive cells were examined using flow cytometry; (**B**–**D**) U-2 OS cells were treated at various temperatures of hyperthermic conditions. After 24 h, the percentage of apoptotic cells was analyzed by conducting flow cytometric analysis on Annexin V/PI double staining. The results are expressed as the mean ± SEM of 4 independent experiments. *, *p <* 0.05 compared with the control group.

To investigate the potential for hyperthermia to induce cell death in human OS cells, we first examined the effect of hyperthermia on cell survival in human OS cells (U-2 OS, MG63 and HOS) using the sulforhodamine B (SRB) assay. The cells with hyperthermia-induced cell death were treated in a temperature-dependent manner (Figure 1A–C). The inhibition of cell proliferation was observed when the cells were exposed with hyperthermia for 60 or 90 min at 43–48 °C. Hyperthermia did not affect the viability of normal bone cells (hFOB 1.19, Figure 1D). We then confirmed that hyperthermia induced cell death through an apoptotic mechanism by performing 4,6-diamidino-2-phenylindole (DAPI) staining, a cell cycle, Annexin V/PI assay, and the terminal deoxynucleotidyl transferase-mediated deoxyuridine triphosphate nick-end labeling (TUNEL) assay. The U-2 OS cells were treated with hyperthermia conditions and, after 24 h, the nuclei of the cells were stained with DAPI (a typical marker of apoptosis). DAPI staining revealed that hyperthermia induced substantial chromatin condensation (Figure 1E). Because continual incubation with hyperthermia caused a substantial reduction in viable cells, we examined the effect of hyperthermia on the induction of cell death in cells by using the cell cycle progression in flow cytometric analysis of propidium iodide (PI) staining. The results shown in Figure 1F–H indicated that hyperthermia induced an increase in the percentage of cells in the sub-G1 phase. In addition, compared with sham-treated U-2 OS cells, hyperthermia-treated cells increased TUNEL fluorescence intensity in a temperature-dependent manner (Figure 2A). We then analyzed whether hyperthermia induced cell death through an apoptotic mechanism. Compared with sham-treated cells, a high proportion of Annexin V labeling was detected in cells treated with hyperthermia (Figure 2B–D). These data indicate that hyperthermia induced cell death through an apoptotic mechanism.

### 2.2. Hyperthermia Induced Mitochondrial Dysfunction and ROS Accumulation

**Figure 3:**
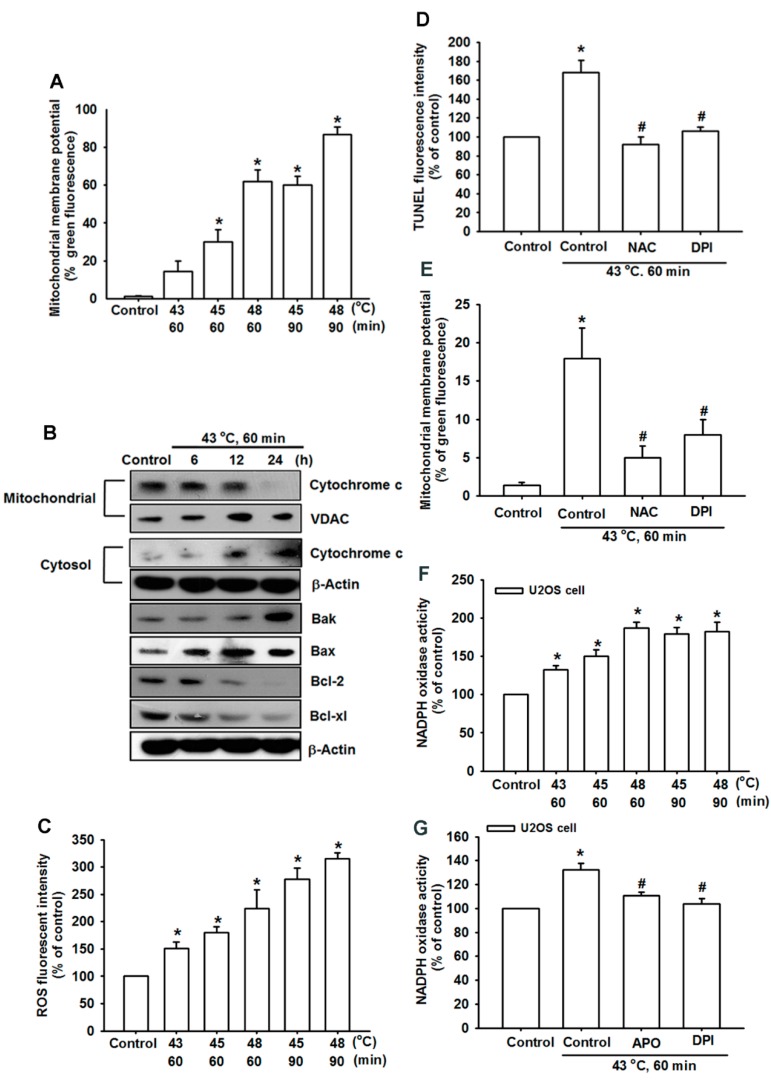
Hyperthermia-induced mitochondrial dysfunction and ROS production in human OS cells. (**A**) U-2 OS cells were treated at various temperatures of hyperthermic conditions. After 24 h, the mitochondrial membrane potential was examined using flow cytometry (*n* = 4); (**B**) U-2 OS cells were incubated with hyperthermia (43 °C, 60 min) for different time intervals and the cytosolic cytochrome c, Bax, Bak, Bcl-2, and Bcl-xL, expressions were examined using Western blot analysis; (**C**) U-2 OS cells were treated at various temperatures of hyperthermic conditions. After 2 h, the production of ROS was examined using flow cytometry (*n* = 4); (**D**) U-2 OS cells were pretreated for 1 h with *N*-acetylcysteine (NAC) and Diphenylene iodonium (DPI), followed by stimulation with hyperthermia (43 °C, 60 min). After 24 h, the percentage of apoptotic cells was then analyzed by conducting flow cytometric analysis on a TUNEL assay; (**E**) U-2 OS cells were pretreated for 1 h with NAC and DPI followed by stimulation with hyperthermia (43 °C, 60 min). After 2 h, the production of ROS was examined via flow cytometry (*n* = 4); (**F**,**G**) U-2 OS cells were treated at various temperatures of hyperthermic conditions or pretreated with APO or DPI and then treated hyperthermia (43 °C, 60 min). After 1 h the NADPH oxidase activity was measured (*n* = 4). The results are expressed as the mean ± SEM. *, *p* < 0.05 compared with the control group. #, *p* < 0.05 compared with the hyperthermia-treated group.

To determine whether hyperthermia induced apoptosis by triggering the mitochondrial dysfunction, we measured the mitochondrial membrane potential by applying a mitochondria-sensitive dye, JC-1 using flow cytometry. As shown in Figure 3A, the treatment of U-2 OS cells with hyperthermia conditions and after 24 h diminished the mitochondrial membrane potential in a temperature-dependent manner. To determine whether hyperthermia induced apoptosis by triggering the mitochondrial apoptotic pathway, we measured changes in the expression of cytochrome c and Bcl-2 family proteins. Mitochondrial and cytosolic proteins were isolated from the hyperthermia-treated U-2 OS cells, and then these proteins were subjected to Western blotting. As shown in Figure 3B, hyperthermia substantially increased cytosolic cytochrome c, and considerably lowered mitochondrial cytochrome c compared with the control group. In addition, the treatment of U-2 OS cells with hyperthermia induced Bax and Bak but reduced Bcl-xL and Bcl-2 levels, causing an increase in the pro-apoptotic/anti-apoptotic Bcl-2 protein ratio (Figure 3B). We then examined whether ROS accumulation was involved in hyperthermia-induced cell apoptosis. The H_2_DCFDA-based FACS detection revealed an increasing level of intracellular H_2_O_2_ in U-2 OS cells after treatment with hyperthermia (Figure 3C). In addition, the pretreatment of cells with *N*-acetylcysteine (NAC; scavenger of ROS) and diphenylene iodonium (DPI; ROS inhibitor) attenuated hyperthermia-induced cell apoptosis (Figure 3D), and attenuated hyperthermia induced the diminishment of the mitochondrial membrane potential (Figure 3E). NADPH oxidase is an important source for the production of ROS [21]. To determine whether ROS was generated by NADPH oxidase, we then examined the effect of hyperthermia on activation of NADPH oxidase in U-2 OS cells. Stimulation of U-2 OS cells increased the NADPH oxidase activity in a temperature-dependent manner (Figure 3E). In addition, pretreatment of cells with APO and DPI attenuated hyperthermia-mediated NADPH oxidase activity (Figure 3F). These data suggest that hyperthermia induced cell apoptosis through mitochondrial dysfunction and ROS accumulation.

### 2.3. Hyperthermia Induced Ca^2+^ Release, GRP78 and Calpain Expression

**Figure 4:**
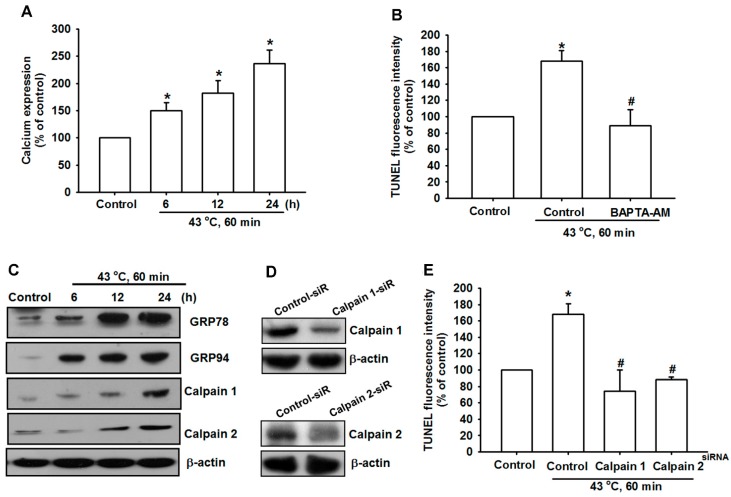
Hyperthermia-induced Ca^2+^ release and ER stress in OS cells. (**A**) U-2 OS cells were incubated with hyperthermia (43 °C, 60 min). After different time intervals, the Ca^2+^ expression was examined using flow cytometry (*n* = 4); (**B**) U-2 OS cells were pretreated for 1 h with BAPTA-AM (10 μM) followed by stimulation with hyperthermia (43 °C, 60 min). After 24 h, the percentage of apoptotic cells was then analyzed by conducting flow cytometric analysis on a TUNEL assay; (**C**) U-2 OS cells were incubated with hyperthermia (43 °C, 60 min). After different time intervals, GRP-78, GRP-94, calpain I, and calpain II expression were examined using Western blot analysis; (**D**,**E**) U-2 OS cells were transfected with calpain I, calpain II, or control siRNA for 24 h before incubation with hyperthermia(43 °C, 60 min). After 24 h, the percentage of apoptotic cells was then analyzed by conducting flow cytometric analysis on a TUNEL assay. The results are expressed as the mean ± SEM. *, *p* < 0.05 compared with the control. #, *p* < 0.05 compared with the hyperthermia-treated group.

To determine whether hyperthermia induced apoptosis by triggering ER stress, we first assessed the effect of hyperthermia on the mobilization of Ca^2+^. When U-2 OS cells were treated with hyperthermia, Ca^2+^ levels increased substantially compared with the sham-treated group. The results indicated that hyperthermia promoted Ca^2+^ release in a time-dependent manner (Figure 4A). By contrast, the pretreatment of cells with BAPTA-AM (Ca^2+^ chelator) reduced hyperthermia-induced cell apoptosis in human OS cells (Figure 4B). The GRP-78 protein is a major ER chaperone, and plays a vital role in regulating ER homeostasis. We examined the effects of hyperthermia on the expressions of GRP-78 and GRP-94 in human OS cells. Hyperthermia markedly increased the levels of GRP-78 and GRP-94 over time (Figure 4C). We then determined whether the expression of calpain would be induced by hyperthermia in OS cells. As shown in Figure 4C, hyperthermia increased calpain I and II expression in a time-dependent manner. Furthermore, the transfection of cells with calpain I or II siRNA substantially reduced hyperthermia-mediated cell apoptosis (Figure 4D–E). Therefore, our data suggest that Ca^2+^ release, as well as GRP-78, GRP-94, and calpain I and II activation were involved in hyperthermia-mediated cell apoptosis.

### 2.4. Hyperthermia Increases Caspase-3 and -9 Expression in U-2 OS Human Osteosarcoma Cells

**Figure 5:**
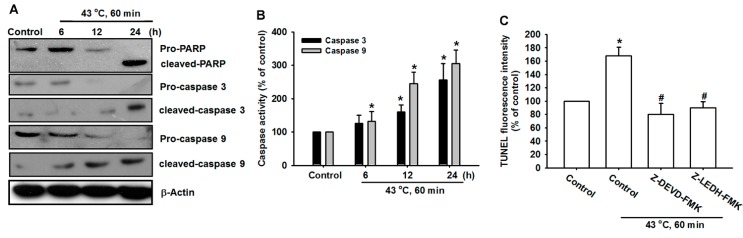
Hyperthermia induces caspase activation in human OS cells. (**A**) U-2 OS cells were incubated with hyperthermia (43 °C, 60 min) for different time intervals, PARP, caspase 3 and caspase 9 expressions were examined using Western blot analysis; (**B**) U-2 OS cells were incubated with hyperthermia (43 °C, 60 min). After 24 h, caspase 3 and caspase 9 activities were examined using a caspase ELISA kit; (**C**) U-2 OS cells were pretreated for 1 h with z-DEVD-FMK (caspase 3 inhibitor) or z-LEHD-FMK (caspase 9 inhibitor), followed by stimulation with hyperthermia (43 °C, 60 min. After 24 h, the percentage of apoptotic cells was then analyzed by conducting flow cytometric analysis on a TUNEL assay. The results are expressed as the mean ± SEM *, *p* < 0.05 compared with the control. #, *p* < 0.05 compared with the hyperthermia-treated group.

ER stress-triggered apoptosis involves the activation of the intrinsic pathway of apoptosis concerning the activity of caspase 3 and caspase 9. Treatment with hyperthermia reduced procaspase 3 expression and increased caspase 3 activity in U-2 OS cells (Figure 5A,B). Hyperthermia increased cleaved-PARP expression (Figure 5A). In addition, upstream procaspase 9 expression decreased when treated with hyperthermia in U-2 OS cells (Figure 5A,B). The pretreatment of cells with the specific caspase 3 inhibitor z-DEVD-FMK and caspase 9 inhibitor z-LEDH-FMK reduced hyperthermia-induced cell death (Figure 5C). These results suggest that hyperthermia triggered caspase activation and induced cancer cell apoptosis in U-2 OS cancer cells.

## 3. Discussion

Although OS is a rare malignant cancer, it is the most common type of malignant bone tumor, and is the eighth most common cancer in childhood malignancies [22]. OS is often treated with chemotherapy, surgery and radiation therapy, and the five-year survival rate for OS remains at 60%–70% [23]. Clinically, a surgical resection remains the primary mode of therapy for OS. Thus, exploring novel and adequate remedies, and developing novel approaches for treating OS is necessary. Hyperthermia has been used for various cancer therapies, but the precise molecular mechanisms remain unclear. Several studies have shown that hyperthermia induced apoptosis in numerous cancer cells [24,25]; however, no report has indicated that hyperthermia affects human OS cells. In this study, we examined the hyperthermia anticancer effect in human OS cells. We determined that hyperthermia inhibited cell viability and induced cell apoptosis in the human OS cell line, but not in the normal bone cell line. In this study, we observed that hyperthermia induced a substantial temperature-dependent induction of OS cells. In addition, we showed that ER stress, mitochondrial dysfunction, ROS accumulation, and caspase activation were involved in hyperthermia-mediated apoptosis in human OS cells.

Mitochondria are responsible for energy production for essential cell functions. Accumulated evidence indicates that mitochondria play a vital role in apoptosis in various cell death paradigms [26]. Mitochondrial dysfunction-induced apoptosis is executed via two major pathways: (1) the release of apoptotic proteins including cytochrome c, apoptosis-inducing factor and DIABLO from the mitochondria into the cytosol [27]; (2) the immediate dissipation of mitochondrial membrane potential and the mitochondrial membrane permeability change [28]. The Bcl-2 family proteins play a pivotal role in various cellular functions, such as cell apoptosis and resistance to conventional chemotherapy [29]. The Bcl-2 family proteins exist in the mitochondrial membrane, and regulate mitochondria-dependent apoptosis with the Bax:Bcl-2 ratio [30]. In this study, we determined that hyperthermia reduced the mitochondrial membrane potential, and increased the release of cytochrome c from the mitochondria into the cytosol. In addition, hyperthermia substantially increased Bax and reduced Bcl-2 expression, suggesting that changes in the ratio of Bax:Bcl-2 contributed to the apoptosis-promotion activity of hyperthermia.

Several reports have presented evidence suggesting that ROS are involved in the regulation of cell death through various stimuli [16,31]. When cells are exposed to apoptotic stimuli, ROS are generated, and they induce mitochondrial permeability transition pore opening, the release of proapoptotic proteins, and the activation of caspase 9 [32]. According to this theory, reducing ROS levels blocks the loss of ΔΨm in a model of activated cell apoptosis. In this study, we determined that hyperthermia mediated oxidative stress by increasing ROS production. The treatment of cells with *N*-acetylcysteine (NAC) and Diphenylene iodonium (DPI) reduced hyperthermia-induced cell death. Our data suggest that ROS accumulation contributed to hyperthermia-induced cell apoptosis in human OS cells.

ER stress is caused by the accumulation of unfolded or misfolded proteins in the ER, and can damage cells [33]. Subjected to various stimuli, the accumulation of unfolded or misfolded proteins in the ER triggers the onset of ER stress, and excessive ER stress triggers cell death [34]. This study determined that hyperthermia induced a number of ER stress markers, including cytosolic calcium level elevation and GRP-78, and GRP-94 activation. Calcium chelator BAPTA-AM blocked hyperthermia-induced cell apoptosis in human OS cells. Overall, these findings indicate that hyperthermia triggered ER stress, as indicated by changes in cytosol calcium levels, which is a critical mediator of hyperthermia-induced cell apoptosis. In conclusion, our data indicated that hyperthermia induced cell death in human OS cells. We showed that hyperthermia induced ER stress, as indicated by changes in cytosol calcium.

## 4. Experimental Section

### 4.1. Materials

Horseradish peroxidase-conjugated antimouse and antirabbit IgG, rabbit polyclonal antibodies specific for cytochrome c, VDAC, Bak, Bax, Bcl-2, Bcl-xL, GRP78, GRP94, calpain I, calpain II, PARP, caspase3, caspase 9 and β-actin (1:1,000 dilution) were purchased from Santa Cruz Biotechnology (Santa Cruz, CA, USA). All of the other chemicals were obtained from Sigma–Aldrich (St. Louis, MO, USA).

### 4.2. Cell Culture and Culture Conditions

The human OS cell line (U-2 OS, MG 63, and HOS) and human fetal osteoblastic cell line (hFOB 1.19) were purchased from the American Type Cell Culture Collection (Manassas, VA, USA). The U-2 OS cells were maintained in McCoy’s 5A medium supplemented with 10% heat-inactivated fetal bovine serum (FBS), and antibiotics (100 U/mL of penicillin and 100 μg/mL of streptomycin) at 37 °C with 5% CO_2_. The MG 63 cells and the HOS cells were maintained in DMEM medium supplemented with 10% heat-inactivated fetal bovine serum (FBS), and antibiotics (100 U/mL of penicillin and 100 μg/mL of streptomycin) at 37 °C with 5% CO_2_. The hFOB 1.19 cells were maintained in a 1:1 mixture of phenol-free DMEM/Ham’s F12 medium (GIBCO-BRL, Gaithersburg, MD, USA) containing 10% heat-inactivated FBS supplemented with 2.5 mM of l-glutamine, 0.3 mg/mL of G418, and antibiotics at 33.5 °C, the permissive temperature for the expression of large T antigens. All of the experiments with the hFOB 1.19 cells were conducted at the permissive temperature of 33.5 °C [35].

### 4.3. Hyperthermia Treatments

The cells were seeded in culture plates under culture conditions overnight before treatment. The plates that were transferred to the oven were pre-heated at the indicated temperatures for the indicated time. For recovery after treatments, the cells were placed in a 37 °C incubator until further analysis [36].

### 4.4. Sulforhodamine B (SRB) Assay

The cytotoxicity of hyperthermia was determined with the SRB assay. The cells were seeded in 200 μL of medium at a concentration of 1 × 10^5^ in 96-well plate, following treatment with hyperthermic conditions. After 1, 2, and 3 days, cells were fixed with 50% trichloroacetic acid to terminate the reaction, and 0.4% SRB in 1% acetic acid was added to each well. After 15 min of incubation, the plates were washed and dyes were dissolved in 10 mM Tris buffer. The 96-well plate was subsequently read by an enzyme-linked immunosorbent assay reader (515 nm) to obtain absorbance density values.

### 4.5. Analysis of Cell Cycle with Flow Cytometry

A quantitative assessment of apoptotic cells was conducted by examining the cell cycle. The cells were collected using centrifugation, and were adjusted to 5 × 10^6^ cells/mL. Prechilled ethanol was added to 0.5 mL of the cell suspensions, and the mixture was incubated at 4 °C for 30 min. Ethanol was then removed using centrifugation, and cellular DNA was stained with a 500 μL propidium iodide (PI) solution (0.1% Triton-X 100, 200 μg/mL of RNase A, and 20 μg/mL of PI in PBS). After staining, the cells were analyzed immediately using the FACScan and CellQuest software (Becton Dickinson, Mountain View, CA, USA). The extent of apoptosis was determined by measuring the DNA content of the cells below a sub-G_1_ peak [37].

### 4.6. Quantification of Apoptosis by Performing Flow Cytometry

Apoptosis was assessed using Annexin V, a protein that binds to phosphatidylserine (PS) residues exposed to the cell surface of apoptotic cells, as previously described [38]. Subsequently, 1 × 10^6^ cells were treated with hyperthermia for the indicated times, washed twice with cold PBS, and resuspended in a staining buffer containing 1 μg/mL of PI and 0.025 μg/mL of Annexin V-FITC. Double-labeling was performed at room temperature for 30 min in the dark, and the cells were immediately analyzed using FACScan and CellQuest (Becton Dickinson) [39].

A quantitative assessment of apoptotic cells was conducted using a TUNEL assay, which is used to examine DNA strand breaks during apoptosis with the BD Apo-Alert™ DNA Fragmentation Assay Kit (Applied Biosystems, Foster City, CA, USA). In brief, the cells were incubated with hyperthermia for the indicated times. The cells were trypsinized and fixed with 4% paraformaldehyde for 15 min at 4 °C. The fixed cells were incubated with digoxigenin-conjugated dUTP in a terminal deoxynucleotide transferase recombinant (rTdT)-catalyzed reaction and nucleotide mixture for 1 h at 37 °C in a humidified atmosphere, and then immersed in a stop/wash buffer for 15 min at room temperature. The stained cells were then analyzed using flow cytometry [40].

### 4.7. 4,6-Diamidino-2-phenylindole (DAPI) Staining

Cells (5000 cells/mL) in 24-well plates were incubated with hyperthermia for the indicated times. Cells in each treatment were individually fixed with 3.7% (*v*/*v*) formaldehyde (Sigma–Aldrich Corp.) for 15 min and then stained by 4,6-diamidino-2-phenylindole (DAPI) for determining cell chromatin condensation. All samples were examined and photographed using Nikon Eclipse TE300 inverted fluorescence microscope (Nikon Corp., Tokyo, Japan) [41].

### 4.8. Determination of the ROS, Production, Ca^2+^ Concentration and Mitochondrial Membrane Potential

Cells were plated in 6-well plates, grown to confluence, and treated with hyperthermia for the indicated times. After incubation, cells were stained with H2DCFDA (10 μM) for ROS determination, JC-1 (5 μg/mL), Fluo 3/AM (3 μg/mL) for Ca^2+^ levels, and JC-1 (5 μg/mL) for ΔΨm. Cells were then immediately analyzed by FACScan and the CellQuest program (Becton Dickinson, Mountain View, CA, USA) [39].

### 4.9. NADPH Oxidase Activity

Cells in 6-well plates were incubated with hyperthermia condition for 60 min. Cells were gently scraped and centrifuged at 400× *g* for 10 min at 4 °C. The cell pellet was resuspended with medium, and the cell suspension was kept on ice. To a final 200 μL volume of medium containing either NADPH (1 μM) or Lucigenin (20 μM), 5 μL of cell suspension (0.2 × 10^5^ cells) were added to initiate the reaction followed by immediate measurement of chemiluminescence in an Appliskan luminometer (Thermo, Finnigan, Austin, TX, USA) in out-of-coincidence mode. Appropriate blanks and control were established, and chemiluminescence was recorded. Neither NADPH nor NADH enhanced the background chemiluminescence of lucigenin alone.

### 4.10. Western Blot Analysis

Cellular lysates were prepared as we described [37]. Proteins were resolved on SDS-PAGE and transferred to Immobilon polyvinyldifluoride membranes. The blots were blocked with 5% BSA for 1 h at room temperature and then probed with rabbit antihuman antibodies against cytochrome c, VDAC, Bak, Bax, Bcl-2, Bcl-xL, GRP78, GRP94, calpain I, calpain II, PARP, caspase 3, caspase 9 and β-actin (1:1000 dilution) for 1 h at room temperature. After being washed three times, the blots were incubated with a peroxidase-conjugated donkey antirabbit secondary antibody (1:1000 dilution) for 1 h at room temperature. The signals were visualized by enhanced chemiluminescence with Kodak X-OMAT LS film (Eastman Kodak, Rochester, NY, USA).

### 4.11. siRNA Transfection

The siRNAs against human calpain I, calpain II and control siRNA were purchased commercially from Santa Cruz Biotechnology. Cells were transfected with siRNAs (at a final concentration of 100 nM) using Lipofectamine 2000 (Invitrogen, Carlsbad, CA, USA) according to the manufacturer’s instructions.

### 4.12. Caspase Activity Assay

The assay is based on the ability of active enzyme to cleave chromophore from enzyme substrate LEHD-pNA (for caspase 9) or Ac-DEVD-pNA (for caspase 3). Cell lysates were prepared and incubated with anticaspase 9 and caspase 3. Immunocomplexes were incubated with peptide substrate in assay buffer (100 mM NaCl, 50 mM 4-(2-hydroxyethyl)-1-piperazine-ethanesulphonic acid (HEPES), 10 mM dithiothreitol, 1mM EDTA, 10% glycerol, 0.1% 3-[(3-cholamidopropyl)dimethylammonio]-1-propanesulfonate (CHAPS), pH 7.4) for 2 h at 37 °C. The release of *p*-nitroaniline was monitored at 405 nm. Results shown are the percent change in activity compared to untreated control.

### 4.13. Statistics

The values reported are means ± SEM. Statistical analysis between two samples was performed using Student’s *t-*test. Statistical comparisons of more than two groups were performed using one-way analysis of variance (ANOVA) with Bonferroni’s *post hoc* test. In all cases, *p* < 0.05 was considered significant.

## 5. Conclusions

This study showed that hyperthermia increased cell death in human OS cells. Hyperthermia induced apoptosis in OS cells by triggering mitochondrial dysfunction, ROS accumulation, and caspase activation. In addition, hyperthermia induced cell death mediated by increasing ER stress, GRP-78, and GRP-94 activation, and Ca^2+^ release, subsequently triggering calpain, caspase 3, and caspase 9 activity, causing apoptosis. Thus, hyperthermia is a promising anticancer approach warranting further development for the treatment of human OS cells.

## References

[1] Krajarng A., Nilwarankoon S., Suksamrarn S., Watanapokasin R. (2012). Antiproliferative effect of α-mangostin on canine osteosarcoma cells. Res. Vet. Sci..

[2] Yang J., Zhang W. (2013). New molecular insights into osteosarcoma targeted therapy. Curr. Opin. Oncol..

[3] Steinmann P., Walters D.K., Arlt M.J., Banke I.J., Ziegler U., Langsam B., Arbiser J., Muff R., Born W., Fuchs B. (2012). Antimetastatic activity of honokiol in osteosarcoma. Cancer.

[4] Tsai H.C., Huang C.Y., Su H.L., Tang C.H. (2014). CCN2 enhances resistance to cisplatin-mediating cell apoptosis in human osteosarcoma. PLoS One.

[5] Tronov V.A., Konstantinov E.M., Kramarenko II. (2002). Hyperthermia induced signal for apoptosis and pathways of its transduction in the cell. Tsitologiia.

[6] Brade A.M., Szmitko P., Ngo D., Liu F.F., Klamut H.J. (2003). Heat-directed suicide gene therapy for breast cancer. Cancer Gene Ther..

[7] Horsman M.R. (2006). Tissue physiology and the response to heat. Int. J. Hyperth..

[8] Nikfarjam M., Muralidharan V., Malcontenti-Wilson C., Christophi C. (2005). Progressive microvascular injury in liver and colorectal liver metastases following laser induced focal hyperthermia therapy. Lasers Surg. Med..

[9] Eddy H.A., Chmielewski G. (1982). Effect of hyperthermia, radiation and adriamycin combinations on tumor vascular function. Int. J. Radiat. Oncol. Biol. Phys..

[10] Falkowska-Podstawka M., Wernicki A. (2003). Heat shock proteins in health and disease. Pol. J. Vet. Sci..

[11] Shellman Y.G., Howe W.R., Miller L.A., Goldstein N.B., Pacheco T.R., Mahajan R.L., LaRue S.M., Norris D.A. (2008). Hyperthermia induces endoplasmic reticulum-mediated apoptosis in melanoma and non-melanoma skin cancer cells. J. Investig. Dermatol..

[12] Pawlik A., Nowak J.M., Grzanka D., Gackowska L., Michalkiewicz J., Grzanka A. (2013). Hyperthermia induces cytoskeletal alterations and mitotic catastrophe in p53-deficient H1299 lung cancer cells. Acta Histochem..

[13] Chen F., Wang C.C., Kim E., Harrison L.E. (2008). Hyperthermia in combination with oxidative stress induces autophagic cell death in HT-29 colon cancer cells. Cell Biol. Int..

[14] Sano R., Reed J.C. (2013). ER stress-induced cell death mechanisms. Biochim. Biophys. Acta.

[15] Toltl L.J., Austin R.C., Liaw P.C. (2011). Activated protein C modulates inflammation, apoptosis and tissue factor procoagulant activity by regulating endoplasmic reticulum calcium depletion in blood monocytes. J. Thromb. Haemost..

[16] Liu J.F., Fong Y.C., Chang K.W., Kuo S.C., Chang C.S., Tang C.H. (2011). FPTB, a novel CA-4 derivative, induces cell apoptosis of human chondrosarcoma cells through mitochondrial dysfunction and endoplasmic reticulum stress pathways. J. Cell. Biochem..

[17] Jiang Y., Lv H., Liao M., Xu X., Huang S., Tan H., Peng T., Zhang Y., Li H. (2012). GRP78 counteracts cell death and protein aggregation caused by mutant huntingtin proteins. Neurosci. Lett..

[18] Bettaieb A., Averill-Bates D.A. (2008). Thermotolerance induced at a fever temperature of 40 degrees C protects cells against hyperthermia-induced apoptosis mediated by death receptor signalling. Biochem. Cell Biol..

[19] Song X., Kim S.Y., Lee Y.J. (2012). The role of Bcl-xL in synergistic induction of apoptosis by mapatumumab and oxaliplatin in combination with hyperthermia on human colon cancer. Mol. Cancer Res..

[20] Venkataraman S., Wagner B.A., Jiang X., Wang H.P., Schafer F.Q., Ritchie J.M., Patrick B.C., Oberley L.W., Buettner G.R. (2004). Over-expression of manganese superoxide dismutase promotes the survival of prostate cancer cells exposed to hyperthermia. Free Radic. Res..

[21] Benavente C.A., Jacobson E.L. (2008). Niacin restriction upregulates NADPH oxidase and reactive oxygen species (ROS) in human keratinocytes. Free Radic. Biol. Med..

[22] Ottaviani G., Jaffe N. (2009). The epidemiology of osteosarcoma. Cancer Treat. Res..

[23] Fromigue O., Hamidouche Z., Vaudin P., Lecanda F., Patino A., Barbry P., Mari B., Marie P.J. (2011). CYR61 downregulation reduces osteosarcoma cell invasion, migration, and metastasis. J. Bone Miner. Res..

[24] Song X., Kim S.Y., Zhou Z., Lagasse E., Kwon Y.T., Lee Y.J. (2013). Hyperthermia enhances mapatumumab-induced apoptotic death through ubiquitin-mediated degradation of cellular FLIP (long) in human colon cancer cells. Cell Death Dis..

[25] Xiao F., Liu B., Zhu Q.X. (2012). c-Jun *N*-terminal kinase is required for thermotherapy-induced apoptosis in human gastric cancer cells. World J. Gastroenterol..

[26] Susin S.A., Zamzami N., Castedo M., Daugas E., Wang H.G., Geley S., Fassy F., Reed J.C., Kroemer G. (1997). The central executioner of apoptosis: Multiple connections between protease activation and mitochondria in Fas/APO-1/CD95- and ceramide-induced apoptosis. J. Exp. Med..

[27] Liu X., Kim C.N., Yang J., Jemmerson R., Wang X. (1996). Induction of apoptotic program in cell-free extracts: Requirement for dATP and cytochrome c. Cell.

[28] Zamzami N., Brenner C., Marzo I., Susin S.A., Kroemer G. (1998). Subcellular and submitochondrial mode of action of Bcl-2-like oncoproteins. Oncogene.

[29] Sturm I., Rau B., Schlag P.M., Wust P., Hildebrandt B., Riess H., Hauptmann S., Dorken B., Daniel P.T. (2006). Genetic dissection of apoptosis and cell cycle control in response of colorectal cancer treated with preoperative radiochemotherapy. BMC Cancer.

[30] Adams J.M., Cory S. (2001). Life-or-death decisions by the Bcl-2 protein family. Trends Biochem. Sci..

[31] Li Z.M., Zhao Y.W., Zhao C.J., Zhang X.P., Chen L.J., Wei Y.Q., Yang H.S. (2011). Hyperthermia increases the therapeutic efficacy of survivinT34A in mouse tumor models. Cancer Biol. Ther..

[32] Lee H., Kim S., Choi B.H., Park M.T., Lee J., Jeong S.Y., Choi E.K., Lim B.U., Kim C., Park H.J. (2011). Hyperthermia improves therapeutic efficacy of doxorubicin carried by mesoporous silica nanocontainers in human lung cancer cells. Int. J. Hyperth..

[33] Han J., Back S.H., Hur J., Lin Y.H., Gildersleeve R., Shan J., Yuan C.L., Krokowski D., Wang S., Hatzoglou M. (2013). ER-stress-induced transcriptional regulation increases protein synthesis leading to cell death. Nat. Cell Biol..

[34] Tsai H.Y., Yang Y.F., Wu A.T., Yang C.J., Liu Y.P., Jan Y.H., Lee C.H., Hsiao Y.W., Yeh C.T., Shen C.N. (2013). Endoplasmic reticulum ribosome-binding protein 1 (RRBP1) overexpression is frequently found in lung cancer patients and alleviates intracellular stress-induced apoptosis through the enhancement of GRP78. Oncogene.

[35] Hou C.H., Lin F.L., Tong K.B., Hou S.M., Liu J.F. (2014). Transforming growth factor alpha promotes osteosarcoma metastasis by ICAM-1 and PI3K/Akt signaling pathway. Biochem. Pharmacol..

[36] Kuo H.T., Chen H.W., Hsiao H.H., Chen H.C. (2009). Heat shock response protects human peritoneal mesothelial cells from dialysate-induced oxidative stress and mitochondrial injury. Nephrol. Dial. Transplant..

[37] Chen J.T., Fong Y.C., Li T.M., Liu J.F., Hsu C.W., Chang C.S., Tang C.H. (2008). DDTD, an isoflavone derivative, induces cell apoptosis through the reactive oxygen species/apoptosis signal-regulating kinase 1 pathway in human osteosarcoma cells. Eur. J. Pharmacol..

[38] Dijkers P.F., Birkenkamp K.U., Lam E.W., Thomas N.S., Lammers J.W., Koenderman L., Coffer P.J. (2002). FKHR-L1 can act as a critical effector of cell death induced by cytokine withdrawal: Protein kinase B-enhanced cell survival through maintenance of mitochondrial integrity. J. Cell Biol..

[39] Liu J.F., Yang W.H., Fong Y.C., Kuo S.C., Chang C.S., Tang C.H. (2010). BFPP, a phloroglucinol derivative, induces cell apoptosis in human chondrosarcoma cells through endoplasmic reticulum stress. Biochem. Pharmacol..

[40] Liu J.F., Huang Y.L., Yang W.H., Chang C.S., Tang C.H. (2012). 1-Benzyl-2-phenylbenzimidazole (BPB), a benzimidazole derivative, induces cell apoptosis in human chondrosarcoma through intrinsic and extrinsic pathways. Int. J. Mol. Sci..

[41] Lin Y.T., Huang A.C., Kuo C.L., Yang J.S., Lan Y.H., Yu C.C., Huang W.W., Chung J.G. (2013). Induction of cell cycle arrest and apoptosis in human osteosarcoma U-2 OS cells by Solanum lyratum extracts. Nutr. Cancer.

